# Heart’s Burden

**DOI:** 10.1038/s41390-025-03839-y

**Published:** 2025-01-15

**Authors:** Tina S. Chai

**Affiliations:** 1161 21st Ave S # D3300, Nashville, TN USA

*Adopted from China at 3 years of age. Had known heart murmur*.

*She was adopted from China as a 4-year-old following Fontan procedure*.

Electronically charted social histories like these caught my attention during my first week rotating in pediatric cardiology. These details caught my attention not just as medical facts but as pieces of a larger, more personal puzzle. It had only been a few days, but a pattern was unfolding. I, an Asian American woman, would introduce myself to American parents whose wide eyes swiftly scanned back-and-forth from their dark-haired daughter to me. At some point, in between my explaining the electrocardiogram and listening for cardiac murmurs, a mother would slide in details of her daughter’s ethnicity and adoption, and I would respond with a quick nod. I did not think much of the child’s stereotypically American last name, or their softer facial features suggestive of Asian ancestry especially when compared to their mother’s Caucasian appearance. I did pay attention, however, to the country that each mother said their child was adopted from: China.

Attending medical school in the South meant rarely seeing children who looked like me. Suddenly, within the first week of this rotation, I had met more Asian American children than in most of my previous rotations combined. Why were there so many girls adopted from orphanages in China? I brought this up with my attending physician, who explained that international adoption had become popular in the Nashville community. She noted that due to China’s previous one-child policy and cultural preferences, a significant number of Chinese girls with complex health conditions, such as heart abnormalities, were placed in orphanages and eventually adopted by American parents. She said there were so many Chinese patients on her panel that a colleague once gifted her an English-to-Chinese dictionary for Christmas.

I suddenly felt sick. My first thought – “How could anyone give up such beautiful children?” – was quickly followed by a second thought “Could that have happened to me?” The one-child policy has always been a part of my life’s backdrop. In elementary school, my parents finally responded to my yearning for a sibling by explaining we were not allowed by law. If we ever wanted to move back to China, they said, we could not have more than one child in the family. In adolescence, I learned that the one-child policy was more than an attempt to slow population growth. It led to significantly more girls given up for adoption than boys due to the cultural preference for sons, contributing to a current female deficit in the Chinese population. From a young age, I understood that, in the country of my mother tongue, there were systemic factors that rendered me less valued simply because of my gender.

Before I was born, my family’s first choice was a son. These were their culturally conditioned beliefs. However, they chose to love me, their only daughter, just the same. After hearing the stories of these adopted girls in cardiology clinic, I do wonder what my future would have looked like had I been diagnosed prenatally with a health condition like hypoplastic left heart or tetralogy of Fallot. If, by whim of biology, my endocardial cushions developed improperly, where would I be?

The one-child policy was implemented from 1980 through 2015. In these 35 years, an estimated 20 million girls “disappeared” from China.^1^ About 120,000 children – mostly girls – left China through international adoption alone, including 85,000 to the United States.^2^ Meeting some of these girls whose lives have been so deeply influenced by this policy, I couldn’t help but think of their early years, spent in orphanages and foster care systems, and the impact these experiences had on their physical and emotional development. Today, they are bright, curious, and kind. As I listen to their hearts during the physical exam, they ask insightful questions about symptoms to look out for and daily habits to maintain good cardiac health. In these children, I see not a reflection of deficiency but a testament to resilience and worth. Despite structurally abnormal hearts, they are just as deserving of an abundance of love and are just as capable of loving wholeheartedly.

*She recently completed second grade. Appeared shy, observed covering her face and head with mom’s shirt which she was using as a blanket. Patient explored feelings about the recent loss of her dog*.

*She has done well since her visit last year. She graduated from high school and will be attending university. She hopes to continue participating in dance while at college*.
